# Bovine leukaemia virus DNA in fresh milk and raw beef for human consumption

**DOI:** 10.1017/S0950268817002229

**Published:** 2017-09-28

**Authors:** N. N. OLAYA-GALÁN, A. P. CORREDOR-FIGUEROA, T. C. GUZMÁN-GARZÓN, K. S. RÍOS-HERNANDEZ, S. P. SALAS-CÁRDENAS, M. A. PATARROYO, M. F. GUTIERREZ

**Affiliations:** 1 PhD Programme in Biomedical and Biological Sciences, Universidad del Rosario, Bogotá, Colombia; 2 Grupo de Enfermedades Infecciosas, Laboratorio de Virología, Departamento de Microbiología, Pontificia Universidad Javeriana, Bogotá, Colombia; 3 Molecular Biology and Immunology Department, Fundación Instituto de Inmunología de Colombia (FIDIC), Bogotá, Colombia; 4 Basic Sciences Department, School of Medicine and Health Sciences, Universidad del Rosario, Bogotá, Colombia

**Keywords:** Bovine leukaemia virus, foodborne infection, fresh milk, raw meat, zoonosis

## Abstract

Bovine leukaemia virus (BLV) is the causative agent of enzootic bovine leucosis, which has been reported worldwide. BLV has been found recently in human tissue and it could have a significant impact on human health. A possible hypothesis regarding viral entry to humans is through the consumption of infected foodstuffs. This study was aimed at detecting the presence of BLV DNA in raw beef and fresh milk for human consumption. Nested PCR directed at the BLV *gag* gene (272 bp) was used as a diagnostic test. PCR products were confirmed by Sanger sequencing. Forty-nine per cent of the samples proved positive for the presence of proviral DNA. This is the first study highlighting the presence of the BLV *gag* gene in meat products for human consumption and confirms the presence of the viral DNA in raw milk, as in previous reports. The presence of viral DNA in food products could suggest that viral particles may also be found. Further studies are needed to confirm the presence of infected viral particles, even though the present findings could represent a first approach to BLV transmission to humans through foodstuff consumption.

## INTRODUCTION

Bovine leukaemia virus (BLV) belongs to the genus *Deltaretrovirus*, family *Retroviridae*, subfamily *Oncovirinae*. This is an oncogenic virus that was first isolated in 1969 and is the aetiological agent of enzootic bovine leucosis (EBL), one of the most frequently occurring neoplastic diseases in cattle [1]; about a third of BLV-infected cows develop persistent lymphocytosis, 1–5% of them developing the late stage of the disease that is associated with B-cell neoplasm [2]. This retrovirus is closely related to the types of human lymphotropic T-cell leukaemia virus (HTLV-1 and -2) [3]. BLV integrates its genome in target bovine cells, so that all infected animals are persistently infected and become carriers of the virus during the course of their lives, thereby having a negative impact on the animals’ immune system and, consequently, induces losses in milk production, poorer yield regarding weight and induced abortions in the animals [1, 4, 5].

A few studies have revealed the presence of DNA and proteins of BLV in human breast tissue samples, proposing that these findings could be considered as a hazardous factor for breast cancer development [6–9]; albeit some different investigations have not found evidence of the virus in people [10, 11], there is proof that a bovine virus is found in human beings, perhaps related to a zoonosis infection. However, it is still not known how BLV is transmitted to humans. Transmission in cattle could be mediated by horizontal or vertical transmission. Vertical transmission includes perinatal transmission through blood or transplacental passage and post-natal infection routes through colostrum and milk consumption [12, 13]. Horizontal transmission could occur by direct contact between infected and non-infected animals, as well as veterinary practices due to using contaminated instruments on many animals without sterilising them between procedures and animals, including tattooing cattle, vaccination and rectal palpations [14]. Conversely, it has been suggested that humans might become infected by consuming foodstuffs from infected animals, through the direct contact involved in livestock practices or vaccines produced with contaminated cattle sera [7, 15]. Although the transmission route has not yet been established, it could be implicated in as-yet-unknown human health issues such as the emergence of new diseases, taking into account that BLV is described as an oncogenic virus.

Food for human consumption has been proposed as a potential source of pathogen transmission. Several viral infections are related to foodborne diseases; enteric viruses, such as rotaviruses and noroviruses, are amongst the viral agents most frequently transmitted by foodstuffs; these agents are transmitted as free viral particles through faecal contamination of foodstuffs for direct consumption, such as fruit and vegetables and (in some exceptions) meat products [16, 17]. Some other viral infections require the presence of infected cells to transmit the agent to other hosts; this is the case of hepatitis A virus and hepatitis E virus (HEV) that have been found in meat products, such as sausages, liver and pork [18–21].

There are other diseases that are, in principle, associated with foodstuff consumption but where the causative agent remains unknown [22]; in spite of most of them being associated with gastrointestinal diseases, there could be other types of pathogens in foodstuffs that are still unknown. This could thus be happening with BLV, involving potential risk for human health. Studies focused on food safety for improving the quality of products prepared for human consumption are needed as foodstuffs could transmit unknown pathogens. This study was thus aimed at evaluating the BLV DNA detection in raw meat and fresh milk (i.e. fresh from milking) for human consumption as a first step in estimating the potential of foodstuffs regarding BLV transmission to humans.

## METHODS

### Sample collection

Convenience sampling was used for obtaining both milk and meat samples, 100 samples were obtained. Fifty beef samples weighing around 15 g each were obtained from butchers in Bogotá whilst the 50 samples of milk were obtained from farms specialising in dairy production located in different parts of Colombia. The milk was collected directly from milking (i.e. before being sent for industrial treatment). The samples were transported to the Virology Laboratory at the Javeriana University in Bogotá where meat samples were stored at −20 °C until being processed, whilst milk samples were processed immediately.

### Sample preparation and nucleic acid extraction

Roche High Pure PCR Template Preparation Kit was used for extracting total nucleic acids from milk and meat, following the manufacturer's indications; some modifications were made for solid tissue and liquid samples. Regarding meat, an initial 10–20 mg of rump cut (muscle) was lysed with proteinase K and the tissue lysis buffer supplied in the DNA extraction kit. Extraction from milk samples first involved cell concentration from an initial 5 ml milk volume through sequential centrifugations at 16 000 ***g*** for 20 min for each cycle (four cycles in total); the pellet so obtained was used for DNA extraction, following the manufacturer's instructions. NanoDrop (Thermo) was used for quantifying the extracted DNA to verify its concentration and purity. The DNA was then frozen (−20 °C) and stored until further use.

### PCR amplification: multiplex and nested PCR

The bovine GAPDH constitutive gene was used as PCR internal control, which was amplified in a multiplex PCR together with the virus’ *gag* gene encoding its capsid proteins. PCR tests were done using Roche PCR master mix with specific primers (0·8 pmol/μl) for the aforementioned genes. Both bovine GAPDH and *gag* primers were previously reported by Buehring *et al*. [6]. An 857 bp fragment was amplified for the bovine GAPDH gene and a 385 bp fragment for *gag*. Multiplex PCR conditions included an initial denaturing step at 94 °C for 5 min, followed by 35 cycles of denaturing at 94 °C for 30 s, an annealing step at 59·3 °C for 60 s and a 90 s extension at 72 °C. A final extension step was performed at 72 °C for 10 min.

PCR sensitivity was increased by nested PCR for samples where the viral gene was not amplified in multiplex PCR. The first PCR's products were used as templates for the nested PCR. The amplification target was an internal *gag* fragment (nt 1097–1369), resulting in a 272 bp fragment (also reported by Buehring *et al*.) [6]. Reaction conditions were the same as those described for multiplex PCR. Annealing temperature was 56 °C with 30 s extension time.

The results for both multiplex and nested PCR were visualised on 1·5% agarose gels prepared in 1× TAE (Biorad) dyed with 1× HydraGreen fluorescent intercalating dye (ACTGene). DNA extracted from a blood sample of an infected animal was used as positive control for BLV and RNase- and DNase-free water as negative amplification control.

### Sequencing

Virus-positive PCR products were purified with PCR product purification kit (Roche High Pure), following the manufacturer's instructions, and then sent to Macrogen Inc. (Seoul, Korea), for Sanger sequencing. The primers (both sense and anti-sense) used for sequencing were the same as those for the nested PCR. BioEdit Sequence Alignment Editor (version 7·2·5) was used for editing and analysing the sequences. Consensus sequences were obtained for each positive sample; the online BLASTn tool was used for verifying the identity of the sequences so obtained.

## RESULTS AND DISCUSSION

BLV has been known and studied as an infectious agent in cattle; however, there are reports of this virus in humans [6, 7, 9] even though the mechanism by which the virus has reached such host has still not been clarified. Buehring *et al*. [15, 23] highlighted three main hypotheses for the viral entry to humans. The first considers direct contact with infected animals; however, such hypothesis would necessarily involve viral presence in limited populations (i.e. veterinarians, livestock handlers and/or farmers). Nevertheless, available evidence has revealed that the virus has been found in people who do not necessarily come into direct contact with animals [6, 7, 9]. The second hypothesis concerns possible BLV transmission through vaccine production processes involving the use of BLV-contaminated foetal bovine sera, even though no experimental evidence has been published regarding this issue. The third hypothesis proposes that the virus might infect humans through the consumption of bovine-derived products from BLV-infected cattle [10, 23], leading to the idea of evaluating meat and milk products for human consumption as a possible pathway for viral entry.

**Fig. 1.**
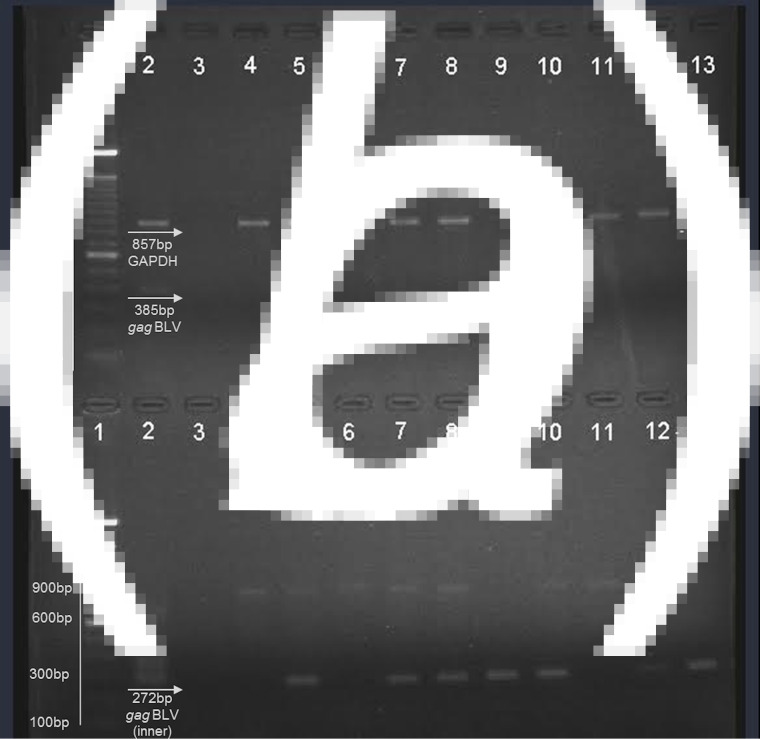
Representative agarose gel (1·5%) of field samples. (*a*) Multiplex PCR. (*b*) Nested PCR. Lane 1 – 100 bp ladder (Invitrogen); lane 2 – positive control; lane 3 – negative control; lane 4–8: beef samples; lane 9–13: milk samples.

Nucleic acids were obtained from fresh milk and raw beef samples (muscle tissues) in the present study in the search for BLV proviral DNA. Internal control (bovine GAPDH) was observed in the multiplex PCR (Fig. 1*a*). A proviral *gag* segment was found in 24 out of the 50 milk samples and in 25 of the meat samples. These results represent 49% of all samples analysed. Most of them were detected by nested PCR, suggesting that the viral load in the samples was considerably low. Figure one shows a representative agarose gel of the results obtained by multiplex PCR (a) where an 857 bp fragment from bovine GAPDH was observed, as well as the external *gag* fragment in positive control (385 bp); nested PCR (b) from the products obtained in the first PCR with a 272 bp fragment was observed in positive control and the samples analysed here (Figs. 1*a* and *b*).

After sequencing PCR products, *gag* gene identity was verified using BLAST (NCBI) with previously reported BLV sequences. The results gave 97–99% identity compared to reference sequences. Such results confirmed that the amplified products obtained from meat and milk samples came from the BLV *gag* segment.

BLV prevalence in Colombia has been recorded as 67·7% on livestock farms throughout the country and in 43% of the bovine population, thereby affecting (health-wise and economically) livestock breeding for milk production and meat for human consumption [24]. Understanding the evolution of the disease in cattle (most infected animals going unnoticed due to low symptomatology) [25] and considering its high prevalence in Colombia highlights the fact that infected animals that have never been detected could be sold for commercialisation, and thus cattle-derived infected products could be distributed in different industries without any government control regarding the presence of the virus. This would favour the disease's dissemination in spite of the fact that EBL was recently established and was considered a disease requiring mandatory notification by the Colombian Agricultural and Livestock Institute [26].

Detection of BLV DNA in cattle-derived foodstuffs (as shown by this study's findings) could serve as a marker, which could suggest a zoonosis (i.e. indicating viral particle transmission by these products). It is worth stressing that cases of zoonosis are considered one of the most important problems regarding infectious disease epidemiology and public health worldwide [27]. Taking the WHO's definition of zoonosis, ‘Any disease or infection that is naturally transmissible from vertebrate animals to humans, including all types of pathogenic agents’, [28] as well as viral transmission mechanisms, action directed towards avoiding viral dissemination in cattle might prevent the introduction of the pathogen into the human population, even though BLV has not yet been conclusively proven to be a cause of human disease [6, 11, 29].

Foodborne diseases are related with ingesting contaminated foodstuffs with microorganisms, which sometimes could come from an animal origin [30]. Two vehicles have been proposed for viral transmission through foodstuff consumption. Free viral particles in foodstuffs has been related to an exogenous contamination source (i.e. faecal contamination), involving direct consumption of fresh products, such as fruit and vegetables [16]. The other possibility concerns the transmission of viruses through animal-derived products infected with the virus. In this case, animal cells would become carriers of pathogenous agents, introducing them into human beings through consumption of meat from infected animals, trespassing even free viral particles, infected cells or proviral DNA [31]. Reports regarding HEV entry to humans have increased due to products from pigs infected by the virus being consumed, i.e. sausages, liver and poorly cooked pork [18, 19, 21, 32]. This situation has led to acute hepatitis outbreaks where a hitherto disregarded zoonosis has been clearly defined. This study's findings have suggested that a similar situation could be occurring with BLV, giving rise to possible explanations for diseases whose causes have previously been undefined.

The pertinent literature has reported viral particles and viral DNA in cows’ milk or colostrum that could be considered a risk factor for transmission to calves [33–36]. Viral DNA was also detected in fresh milk in the present study, thereby agreeing with previous reports, despite not having been described as a risk factor for humans. Bearing this in mind, some other retroviruses could be transmitted by their respective hosts consuming milk, i.e. HTLV, MMTV (mammary murine tumor virus) and also HIV in particular situations [37–39].

Consuming raw milk could be a viable transmission pathway, mostly in developing countries having high raw milk consumption in rural populations. Previous studies have established that industrialisation of milk and pasteurisation processes leads to inactivating viral particles [40–42]; avoiding raw milk consumption would thus be an essential prevention strategy, even if it remains unknown whether BLV can also reach humans by this means.

It is worth highlighting that even though the study's objective was not to determine the presence of complete and infectious viral particles in the samples analysed, the gene fragments found here suggested the virus could be found, since comparing the sequences obtained here with previously reported ones gave 97–99% identity (i.e. dealing with BLV). Further studies should be aimed at establishing whether consuming the aforementioned foodstuffs transmits infective viral particles, which can then complete their biological cycle in humans. It could be of great interest to evaluate viral presence after cooking meat as this could inactivate viral particles and to ascertain whether other mechanisms could be participating in viral transmission [40].

Moreover, this is the first experimental approach reporting the BLV *gag* gene segment being detected in beef products for human consumption. Questions concerning viral transmission through consuming infected meat have been raised since the reports by Buehring *et al*. [15]. However, only empirical approaches and inferences about this transmission pathway have been proposed, regarding slaughterhouse practices involving carcinogenic cattle tissue where not only these tissues are distributed to humans, but have been disposed of for dog and cat food products [43]. The data reported here are important for foodborne infections and public health. Prevention policy, which proposes the early detection of pathogenous agents with a possibility to reach humans, depends on the risks for the target population, by controlling the main sources of dissemination [44].

The presence of the BLV DNA in bovine-derived products could be interpreted as a step forward in identifying previously unknown foodborne diseases. Our results suggested that BLV could be considered a potential zoonotic agent, even though non-infectious particles were reported in this study. Evidence of an oncogenic virus’ DNA in milk and meat products highlights such foodstuffs as being a potential source of viral transmission to humans and could be the outcome of currently unknown diseases. Such viruses’ alternative transmission routes should be studied (i.e. human-to-human transmission). Prevention and control strategies should be enforced to decrease viral prevalence and transmission in cattle and ensure that infected foodstuffs do not become distributed to markets; such alternatives aimed at eradicating the disease have been achieved in some European countries, New Zealand and Australia [5, 45].
